# Case Report: Autoimmune Lymphoproliferative Syndrome vs. Chronic Active Epstein-Barr Virus Infection in Children: A Diagnostic Challenge

**DOI:** 10.3389/fped.2021.798959

**Published:** 2021-12-30

**Authors:** Aleksandra Szczawińska-Popłonyk, Elzbieta Grześk, Eyal Schwartzmann, Anna Materna-Kiryluk, Jadwiga Małdyk

**Affiliations:** ^1^Department of Pediatric Pneumonology, Allergology and Clinical Immunology, Institute of Pediatrics, Poznań University of Medical Sciences, Poznań, Poland; ^2^Department of Pediatrics, Hematology and Oncology, Nicolaus Copernicus University, Bydgoszcz, Poland; ^3^English Division, Poznan University of Medical Sciences, Poznań, Poland; ^4^Department of Medical Genetics, Poznan University of Medical Sciences, Poznań, Poland; ^5^Department of Pathology, Medical University of Warsaw, Warsaw, Poland

**Keywords:** autoimmune lymphoproliferative syndrome, chronic active Epstein-Barr virus infection, lymphoproliferation, children, immunodeficiency–primary

## Abstract

Autoimmune lymphoproliferative syndrome (ALPS) is a disorder characterized by a disruption of the lymphocyte apoptosis pathway, self-tolerance, and immune system homeostasis. Defects in genes within the first apoptosis signal (FAS)-mediated pathway cause an expansion of autoreactive double-negative T cells leading to non-malignant lymphoproliferation, autoimmune disorders, and an increased risk of lymphoma. The aim of the study was to show the diagnostic dilemmas and difficulties in the process of recognizing ALPS in the light of chronic active Epstein-Barr virus (CAEBV) infection. Clinical, immunological, flow cytometric, biomarkers, and molecular genetic approaches of a pediatric patient diagnosed with FAS-ALPS and CAEBV are presented. With the ever-expanding spectrum of molecular pathways associated with autoimmune lymphoproliferative disorders, multiple genetic defects of FAS-mediated apoptosis, primary immunodeficiencies with immune dysregulation, malignant and autoimmune disorders, and infections are included in the differential diagnosis. Further studies are needed to address the issue of the inflammatory and neoplastic role of CAEBV as a triggering and disease-modifying factor in ALPS.

## Introduction

Autoimmune lymphoproliferative syndrome (ALPS) also known as Canale-Smith syndrome is a disorder of the extrinsic first apoptosis signal (FAS)-mediated pathway of lymphocyte apoptosis characterized by marked genetic and clinical heterogeneity (1, 2). The hallmarks of the disease are childhood-onset chronic non-malignant, non-infectious lymphoproliferation manifesting as lymphadenopathy and/or splenomegaly, autoimmune cytopenia, and polyclonal IgG hypergammaglobulinemia. The defective lymphocyte apoptosis gives rise to non-malignant lymphoid hyperplasia with an expansion and an accumulation of autoreactive T cells which, in turn, triggers autoimmune phenomena. Clinical symptomatology in children most frequently includes multilineage cytopenia, but the involvement of solid organs such as hepatitis, uveitis, systemic lupus erythematosus, Guillain-Barre syndrome, and glomerulonephritis have also been noted. Affected individuals show a significantly increased risk for the development of malignancies, such as B-cell Hodgkin lymphoma, non-Hodgkin lymphoma, leukemia, or solid tumors of the breast, liver, and thyroid gland (3).

In ALPS, the immunopathology is associated with the FAS [tumor necrosis factor receptor superfamily 6 (TNFRSF6), CD95, APO1]-mediated process of activation-induced cell death (AICD). Upon T lymphocyte activation, an expression of Fas ligand (FASL) and its interaction with FAS receptors is triggered, leading subsequently to binding of FAS-associated protein with death domain (FADD). FADD then recruits pro-caspases 8 and 10 to engender the death-inducing signaling complex (DISC) and generates terminal caspase activation leading to cell death (1–3). This tightly regulated system of controlling lymphocyte stimulation plays a critical role in immune homeostasis thereby preventing an expansion of autoreactive T cells. Abnormal FAS-mediated activation-induced apoptosis pathway is caused by germline or somatic mutations in *FAS* which is the main genetic defect in the majority of ALPS patients (2, 4), *FASLG, CASP8*, and *CASP10* genes, encoding crucial elements of AICD, Fas cell surface death receptor protein, FAS ligand protein, and caspases 8 and 10, respectively (5). While widely considered to be a primary immune deficiency presenting in early childhood, an ever-growing understanding of the immunogenetic background of ALPS has led to increasingly recognized adult-onset and ALPS-like disorders with mutations in *NRAS* and *KRAS* genes as well (5). The advances in ALPS delineation and definition have also been summarized by international investigators at the National Institutes of Health (NIH) in 2009, leading to the novel disease classification engaging genetic information and biomarkers (6). According to the revised nomenclature incorporating genetic variants, patients fulfilling the diagnostic criteria and having germline homozygous or heterozygous mutation in *FAS*, somatic mutations in *FAS*, germline mutations in *FASLG*, and germline mutations in *CASP10* have been defined as ALPS-FAS, ALPS-sFAS, ALPS-FASLG, and ALPS-CASP10, respectively, while patients with the undetermined genetic defect have been classified as ALPS-U (6). Furthermore, a group of ALPS-related disorders has also been defined based on clinical symptomatology, diagnostic tests, and genetic variants in *CASP8, NRAS, SH2D1A* corresponding with CEDS (caspase 8 deficiency state), RALD (RAS-associated autoimmune leukoproliferative disease), XLP1 (X-linked lymphoproliferative syndrome), as well as DALD (Dianzani autoimmune lymphoproliferative disease) with unknown genetic background (6, 7). The proposed revised diagnostic criteria for ALPS have embraced extended clinical, immunological, histopathological features to maximize the accurateness of the diagnosis. The relative count of CD3+TCRαβ+CD4-CD8- double-negative (DN) T cells among total lymphocytes and CD3+ lymphocytes, defective lymphocytes apoptosis test, and elevated plasma sFASL levels have been underscored, and a combination of those two last-mentioned criteria proved to be the most important prognosticators for patients with *FAS* mutation (8). In 2019, ESID Working Definitions for Clinical Diagnosis of ALPS licensed a wider range of combinations of clinical and biomarker criteria with equal connotation and not including the ALPS genetic diagnosis (9). Nevertheless, sequential decisions on the multistep diagnostic process guided by NIH or ESID criteria leading to establishing a definitive ALPS diagnosis are still challenging and require a thorough differential diagnosis. Marked overlap of the clinical symptomatology with other disorders of immune dysregulation, namely primary immunodeficiencies, such as CVID (common variable immunodeficiency), RAG1 (recombination activation gene 1), IKBKG (inhibitor of nuclear factor kappa B (NF-κB) kinase regulatory subunit gamma) or NEMO (NF-κB essential modulator), STAT3 GOF (signal transducer and activator of transcription 3 gain-of-function), LRBA (Lipopolysaccharide (LPS) responsive beige-like anchor protein), CTLA-4 (Cytotoxic T lymphocyte antigen 4), MYO5B (myosin Vb) defects (3, 7, 10–14), primary or secondary HLH (hemophagocytic lymphohistiocytosis) as well as systemic lymphoproliferative disease in chronic active Epstein-Barr virus (EBV) infection (CAEBV) may confound the clinical diagnosis (15).

## Aim of the Study

To expand the clinical and immunological phenotype of a pediatric patient showing a unique association of ALPS and CAEBV. We also aimed at showing the challenges of the clinical observations and difficulties in the diagnostic workup of ALPS with a concomitant EBV infection.

## Patient and Methods

### The Patient

A 5-year-old boy was referred to our pulmonology, allergy, and clinical immunology unit of the pediatric university hospital with a suspicion of primary immunodeficiency due to pancytopenia, splenomegaly, and lymphadenopathy.

He was born to a young, healthy, non-consanguineous couple, at 39 weeks gestational age (WGA), by cesarean section, in a good general condition, with an Apgar score of 10 and birth weight of 4310 g. During the first year of life, his psychomotor development was normal, he did not suffer from infections. He received all live attenuated (BCG after birth and measles/mumps/rubella in the 13th month of life) and inactivated vaccines (against poliomyelitis, pertussis, tetanus, 10-valent polysaccharide conjugated pneumococcal vaccine), without adverse effects following immunization (AEFI). At the age of 2 years, he was hospitalized in a regional pediatric hospital due to pneumonia with thrombocytopenia 100 × 10$\hat{3}$/mcL, splenomegaly (length 14.4 cm), and positive EBV anti-VCA IgM. After 3 months he presented hepatosplenomegaly and petechiae accompanied by pancytopenia: the platelet count decreased to 70 × 10$\hat{3}$/mcL, anemia, leukopenia with neutropenia and eosinophilia were also observed. At that time, the diagnostic workup included: infections (serological tests against CMV, HCV, *Toxocara canis, Toxoplasma gondii, Ascaris lumbricoides*), autoimmune disorders (antiglobulin Coombs tests, ANA, ANCA autoantibodies), and metabolic diseases (Niemann-Pick A/B, Gaucher disease), all negative. Due to the suspicion of a malignant process, the boy was referred to the hematology and oncology unit of our university hospital, where a bone marrow aspiration was performed and features of malignant lymphoproliferation were not detected. Further monitoring of the patient's hematological parameters by a specialist in pediatric hematology/oncology for the next almost 3 years in ambulatory care revealed persistent splenomegaly and lymphadenopathy with pancytopenia (Supplementary Figure 1).

### Methods

Clinical evaluation and a multidirectional diagnostic workup were performed in the patient aged 5 years old, including:

1. In-depth pediatric history concerning the onset of lymphadenopathy and the number of groups of enlarged lymph nodes as well as the onset of splenomegaly together with the physical examination to enable assessment of these crucial clinical data according to the NIH and ESID diagnostic criteria for ALPS
2. Thoracic X-ray and abdominal ultrasound examination to assess mediastinal and abdominal lymphadenopathy along with splenomegaly
3. Analysis of basic hematology and biochemistry, with the complete blood count (CBC), differential white blood count (WBC), ferritin, lactic dehydrogenase (LDH) activity, fasting triglycerides (TG), cholesterol, and fibrinogen
4. Advanced immunology including serum immunoglobulin G, M, and A levels, flow cytometric analysis of B and T cell subsets with the particular emphasis on evaluation of the relative and absolute counts of CD3+CD4-CD8- double-negative (DN) T cells, which constitute an important NIH and ESID diagnostic criterion for ALPS
5. Screening for markers of autoimmunity: indirect and direct Coombs test, antinuclear (ANA) and antineutrophil cytoplasmic (ANCA) antibodies, anti-thyroid antibodies
6. Serological tests and RT-PCR identification of viral and bacterial etiology of symptoms as an element of the differential diagnosis
7. Evaluation of biomarkers recommended both by NIH and ESID as diagnostic predictors for ALPS: cobalamin (vitamin B12), interleukin (IL)-10
8. Lymph node biopsy for immunohistological findings of ALPS
9. The molecular genetic testing of 11 genes contained in Invitae ALPS Panel

## Results

The patient presented clinical symptoms of chronic, more than 3-year-lasting lymphoproliferation, with splenomegaly and cervical, submandibular, retroauricular, supra- and subclavicular, axillar, and inguinal lymphadenopathy, accompanied by enlarged abdominal lymph nodes forming a conglomerative mass assessed in the ultrasound examination. The spleen reached maximal diameters 17.2 × 6.6 cm (length × width), corresponding with the 3rd degree of splenomegaly according to Hackett's WHO grading system for palpable examination. In peripheral blood, a variable decrease of all cell lineages, with hemoglobin ranging from 7.1 to 9.9 g/dL, RBC from 2.75 to 3.89 × 10$\hat{6}$/mcL, WBC from 1.51 to 3.02 × 10$\hat{3}$/mcL, and PLT count from 71 to 86 × 10$\hat{3}$/mcL and neutropenia from 0.1 to 0.48 × 10$\hat{3}$/mc were noted. Both serum screening tests useful in diagnosing malignancy, LDH activity and ferritin level were not increased, as well as fasting TG, cholesterol, and plasma fibrinogen levels as screening biochemical tests toward hemophagocytic lymphohistiocytosis (HLH) were normal.

**Figure 1:**
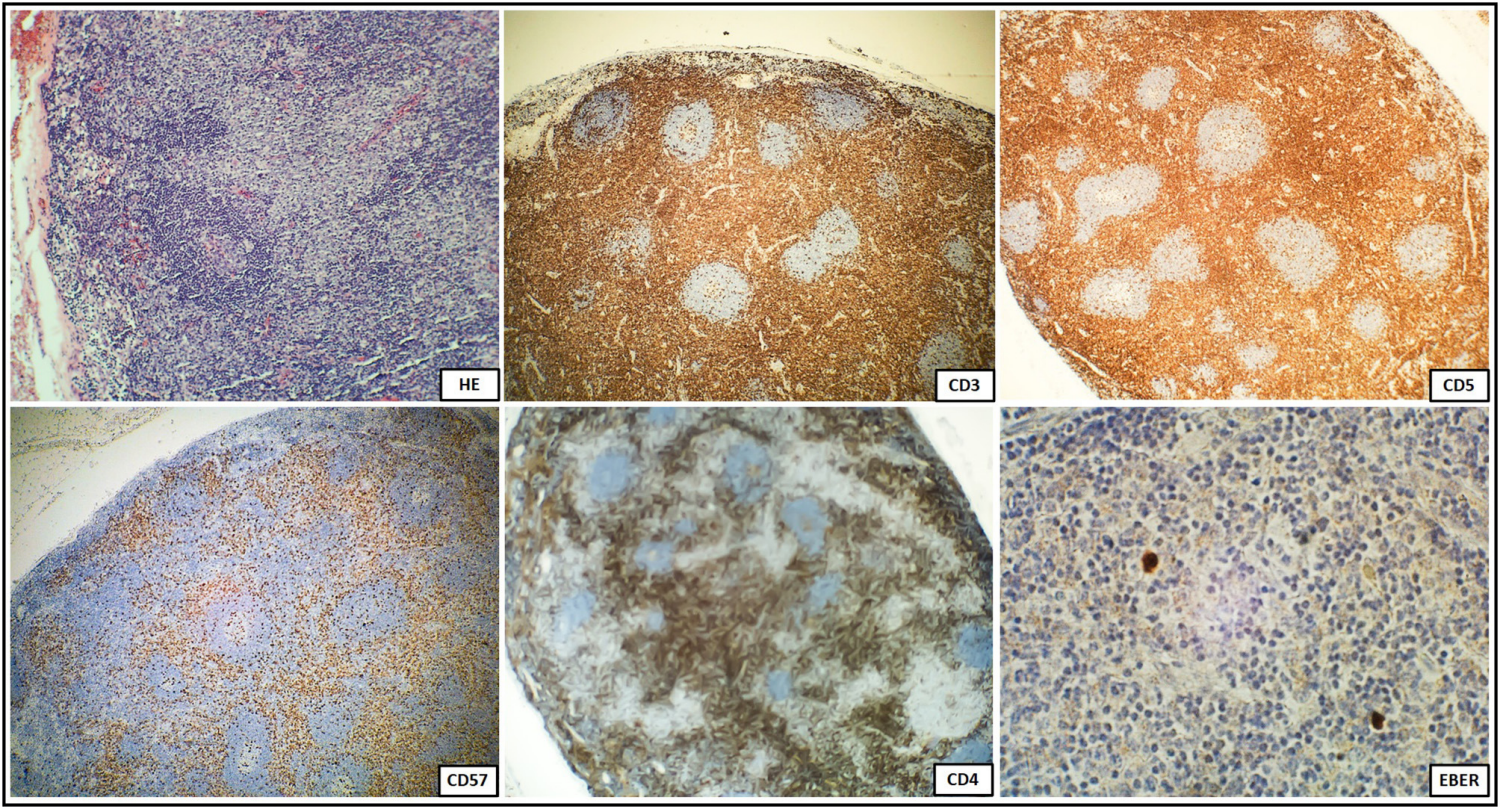
Immunohistochemical staining of a biopsied lymph node with regressive lumps showing diffusely positive CD3+CD57+CD5+NK cells and numerous reactive CD4 positive T cells demonstrating a high proliferation index, and sparse EBER positive cells.

Concerning for ALPS, immunological tests were performed according to the suggested NIH algorithm, and showed polyclonal IgG hypergammaglobulinemia, elevated relative and absolute TCR alpha/beta CD3+CD4-CD8- DN T cells among total and CD3+ lymphocytes along with markedly elevated serum levels of the most discriminatory biomarkers, cobalamin and IL-10. A summary of the clinical evaluation and laboratory workup of the patient, according to the NIH and ESID diagnostic criteria are displayed in Table 1. The recommended FAS-mediated apoptosis assay and soluble FASL blood level were not examined due to the lack of availability of these tests. The flow cytometric analysis of the B and T cell peripheral blood compartments is displayed in Table 2. Because of the high index of suspicion, molecular genetic testing of 11 genes included in Invitae ALPS Panel was performed and showed a germline heterozygous pathogenic mutation in *FAS*, consistent with the diagnosis of ALPS-FAS. The panel of genes analyzed and a description of the variant found and its pathogenicity are shown in Table 3. Of note, neither the presumed autoimmune etiology of anemia or neutropenia was documented nor ANA, ANCA, anti-tissue transglutaminase (anti-tTG), or anti-thyroid peroxidase (anti-TPO), anti-thyroglobulin (anti-TG), and anti-thyrotrophin (TSH) (anti-TR) antibodies were detected. The results of the differential diagnostics are displayed in Table 4. In search of results supporting the ALPS diagnosis, bone marrow aspirate microscopic and flow cytometric evaluation were done, which, besides the increased number of CD3+HLA-DR+ activated T cells, did not show impaired development of lymphocytic, myeloid, and erythroid lines or DN TCR alpha/beta T cells. While recommended by NIH, histopathological examination of the lymph node was performed, not indicating for ALPS or HLH, but of CAEBV NK-cell type. In the biopsied lymph node, regressive lumps showing proliferation of atypical CD3+ CD57+ CD5+ NK cells in the deep cortex and numerous reactive T helper CD4+ lymphocytes were found. The expression of the nuclear Ki67 antigen, a marker of proliferation, was present in 70–80% of cells with the Epstein Barr virus-encoded small RNAs (EBERs) seen in sparse cells (Figure 1).

**Table 1:** Clinical and laboratory workup according to the revised 2009 NIH and 2019 ESID diagnostic criteria for ALPS.

**Revised 2009 NIH diagnostic criteria for ALPS**	
**Required (NIH criteria)**	**Required (the patient)**
Chronic (>6 months), non-malignant, non-infectious lymphadenopathy or splenomegaly or both Elevated CD3+TCRαβ+CD4-CD8- DNT cells (>1.5%) of total lymphocytes and (>2.5%) of CD3+ lymphocytes in the setting of normal or elevated lymphocyte counts	Chronic (>3 years), non-malignant lymphadenopathy and splenomegaly Elevated CD3+TCRαβ+CD4-CD8- DNT cells 4.6–8.3% of total lymphocytes and 6.7–10.2% of CD3+ lymphocytes in the setting of normal lymphocyte counts (1950cc−2503cc)
**Accessory (NIH criteria)**	**Accessory (the patient)**
**Primary** 1. Defective lymphocyte apoptosis (in two separate assays) 2. Somatic or germline pathogenic mutation in *FAS, FASLG*, or *CASP10* **Secondary** 1. Elevated plasma sFASL levels (>200 pg/mL) or elevated plasma IL-10 levels (>20 pg/mL) or elevated serum or plasma vitamin B12 levels (>1200 ng/L) or elevated plasma IL-18 levels >500 pg/mL 2. Typical immunohistological findings as reviewed by an experienced hematopathologist 3. Autoimmune cytopenia (hemolytic anemia, thrombocytopenia, or neutropenia) and elevated immunoglobulin G levels (polyclonal hypergammaglobulinemia) 4. Family history of a non-malignant/non-infectious lymphoproliferation with or without autoimmunity A definitive ALPS-FAS diagnosis is based on the presence of both required criteria plus one primary accessory criterion. Additionally, two secondary accessory criteria support the definitive diagnosis	**Primary** 1. Nd 2. Germline c.749G>A (p.Arg250Gln) pathogenic mutation in *FAS* **Secondary** 1. Elevated plasma biomarkers: IL-10 serum level 160 pg/mL, vitamin B12 serum level 3726 pg/mL, IL-18 serum level nd, plasma sFASL level nd 2. Typical immunohistological findings for ALPS as reviewed by an experienced hematopathologist not present 3. Autoimmune cytopenia (HGB 7.1 g/dL, HCT 20.7%, RBC 2.75 × 10$\hat{6}$/mcL, WBC 3.02 × 10$\hat{3}$/mcL, PLT 71 × 10$\hat{3}$/mcL, neutropenia 0.48 × 10$\hat{3}$/mcL 4. and polyclonal hypergammaglobulinemia IgG 1680 mg/dL−1882 mg/dL (N: 570–1410 mg/dL)
**ESID 2019 working definitions for clinical diagnosis of ALPS (criteria not including genetic diagnosis)**	
**At least one of the following (ESID criteria):** •Splenomegaly •Lymphadenopathy (>3 nodes, >3 months, non-infectious, non-malignant) •Autoimmune cytopenia (>/= 2 lineages) •History of lymphoma •Affected family member **And at least one of the following (ESID diagnostic marker):** •TCRab+CD3+CD4-CD8- of TCRab+CD3+ T cells >6% •Elevated biomarkers (at least two of the following): •sFASL >200 pg/mL •Vitamin B12 >1500 ng/L •IL-10 >20 pg/mL •Impaired FAS mediated apoptosis	**Clinical criteria (the patient):** •Splenomegaly •Lymphadenopathy >20 nodes, >3 years, non-malignant •Autoimmune cytopenia: anemia, leukopenia, thrombocytopenia, neutropenia •No history of lymphoma or family members affected **And at least one of the following diagnostic marker (the patient):** •TCRab+CD3+CD4-CD8- of TCRab+CD3+ T cells 6.7–10.2% •Elevated biomarkers: •sFASL nd •Vitamin B12 3726 pg/mL •IL-10 160 pg/mL •FAS-mediated apoptosis nd

**Table 2:** Peripheral blood flow cytometric immunophenotyping.

**Lymph cell subset**	**WBC 3370cc**	**WBC 5890cc**	**Reference values**
Lymphocytes CD45+/SSC low	58%, 1950cc	43%, 2503cc	29–46%, 1400–5500cc
B CD19+	10%, 209cc	9%, 224cc	8–39%, 180–1300cc
Transitional B CD19+CD38+IgM++	23.2%, 48cc	18.4%, 41cc	3.1–12.3%, 20–200cc
Mature na?ve B CD19+CD27-IgD+	80.0%, 167cc	71.0%, 159cc	54.0–88.4%, 280–1330cc
Non-switched memory B (MZL) CD19+CD27+IgD+	10.7%, 22cc	8.0%, 20cc	2.7–19.8%, 20–180cc
Switched memory B CD19+CD27+IgD-	6.3%, 13cc	5.0%, 11cc	7–21.2%, 20–220cc
Immature B CD19+CD21lo	9.1%, 19cc	9.8%, 22cc	4.1–24.4%, 20–230cc
Activated B CD19+CD38loCD21lo	5.5%, 11cc	8.2%, 18cc	1.7–5.4%, 10–60cc
Plasmablasts CD19+CD38++IgM-	0.0%, 0cc	0.0%, 0cc	0.6–4.0%, 10–50cc
T CD3+	74.0%, 1491cc	77.0%, 1947cc	52–92%, 850–4300cc
T CD3+CD4-CD8-TCR alpha/beta in lymph	4.6%, 99cc	8.3%, 207cc	<1%
T CD3+CD4-CD8-TCR alpha/beta in T lymph	6.7%, 100cc	10.2%,199cc	<1.5%
T helper CD3+CD4+	27.0%, 534cc	28%, 719cc	25–66%, 500–2700cc
T suppressor/cytotoxic CD3+CD8+	34.0%, 674cc	34%, 854cc	9–49%, 200–1800cc
CD4+/CD8+	0.79	0.84	1.5–2.5
Recent thymic emigrants CD3+CD4+CD45RA+CD31+	57.0%, 304cc	63.5%, 457cc	37–100%, 190–2600cc
Naïve T helper CD3+CD4+CD45RA+CD27+	66.1%, 353cc	71.3%, 513cc	52–92%, 300–2300cc
Central memory T helper CD3+CD4+CD45RA-CD27+	23.9%, 127cc	19.5% 140cc	15–56%, 160–660cc
Effector memory T helper CD3+CD4+CD45RA-CD27-	9.6%, 51cc	5.7%, 41cc	0.3–9%, 3–89cc
Terminally differentiated memory T helper CD3+CD4+CD45RA+CD27-	0.4%, 2cc	3.5%, 25cc	0–1.2%, 0.0–16cc
Follicular CXCR5+ T helper CD3+CD4+CD45RO+CD185+	26.0%, 51cc	32.8%, 60cc	6–72%, 13–170cc
Regulatory T helper CD3+CD4+CD25++CD127-	0.5%, 3cc	1.6%, 11cc	3–17%, 39–150cc
Naïve T suppressor/cytotoxic CD3+CD8+CD27+CD197+	27.7%, 187cc	32.1%, 274cc	19–100%, 53–1100cc
Central memory T suppressor/cytotoxic CD3+CD8+CD45RA-CD27+CD197+	1.2%, 8cc	1.4%, 12cc	1–9%, 4–64cc
Effector memory T suppressor/cytotoxic CD3+CD8+CD45RA-CD27-CD197-	7.4%, 50cc	10.3%, 88cc	10–55%, 24–590cc
Terminally differentiated T suppressor/cytotoxic CD3+CD8+CD45RA+CD27-CD197-	3.1%, 216cc	12.6%, 108cc	6–83%, 25–530cc
NK CD3-CD45+CD16+CD56+	5.0%, 106cc	8.0%, 191cc	2–25%, 61–510cc

**Table 3:** Genetic testing comprising 11 genes involved in immune dysregulation disorders.

**Sequence analysis and deletion/duplication testing of 11 genes (ALPS panel)**	
**Genes analyzed**	***FAS*** **variant found in the patient**
*FAS* (Fas Cell Surface Death Receptor)	Heterozygous pathogenic c.749G>A (p.Arg250Gln), aka R234Q *FAS* variant
*FASLG* (Fas Ligand)	**Pathogenicity of the** ***FAS*** **variant in the patient**
*FADD* (Fas-Associated Death Domain)	The sequence change replaces arginine with glutamine at codon 250 of the FAS protein. The arginine residue is highly conserved and there is a small physicochemical difference between arginine and glutamine. Advanced modeling of protein sequence and biophysical properties (such as structural, functional, and spatial information, amino acid conservation, physicochemical variation, residue mobility, and thermodynamic stability) indicates that this missense variant disrupts the p.Arg250 amino acid residue in FAS and affects FAS protein function, and accordingly, this variant has been classified as pathogenic
*CASP10* (Caspase 10)	
*CASP8* (Caspase 8)	
*CTLA4* (Cytotoxic T Lymphocyte Antigen 4)	
*ITK* (IL-2 Inducible T Cell Kinase)	
*MAGT1* (Magnesium Transporter 1)	
*PIK3CD* (Phosphatidylinositol-4,5 Biphosphonate 3-Kinase Catalytic Subunit Delta)	
*PRKCD* (Protein Kinase C Delta)	
*STAT3* (Signal Transducer and Activator of Transcription3)

**Table 4:** Differential diagnosis of ALPS and overlapping lymphoproliferative conditions.

**Differential diagnosis**
**Infections**
EBV: VCA IgM 0.03 s/co negative, VCA IgG 50.73 s/co positive, EBNA IgG 9.65 s/co positive, EBV RT-PCR 554–1526 DNA copies/mL CMV, VZV, *Enterovirus, Adenovirus, Parechovirus*, hPVB19, HSV1,2, HHV6,7 RT-PCR in PB negative *Influenza virus* A, AH1N1, B, *Coronavirus* NL63, 229E, OC43, HKU1, *Parainfluenza virus* 1,2,3,4, *Metapneumovirus* A, B, *Bocavirus, Rhinovirus*, RSV A, B, *Adenovirus, Enterovirus, Parechovirus, Mycoplasma pneumoniae, Chlamydophila pneumoniae, Streptococcus pneumoniae, Staphylococcus aureus* RT-PCR in nasopharyngeal aspirate negative QuantFERON-TB: endogeneous IFN-γ 0.08 IU/mL, IFN-γ release by CD4+ T cells: 0.05 IU/mL, IFN-γ release by CD8+ T cells: 0.05 IU/mL, PHA-stimulated IFN-γ release: >10 IU/mL; negative *Bartonella henselae*: IgG negative 6. *Toxocara canis*: IgG negative 7. HIV: anti-HIV1, HIV2, p24 negative 8. SARS-CoV2 PCR negative
**Autoantibodies**
Coombs direct antiglobulin test: anti-IgG, anti-IgM, anti-IgA, anti-C3c, anti-C3d negative Anti-TG 2.3 IU/mL, anti-TPO <1.0 IU/mL, anti-TR negative Anti-tTG IgA, IgG negative ANA: nRNP/Sm, Sm, SS-A, Ro-52, SS-B, Scl-70, PM-Scl100, Jo-1, CENP B, PCNA, dsDNA, nucleosomes, histones, ribosomal protein P, AMA-M2 negative ANCA: proteinase-3, lactoferrin, myeloperoxidase, elastase, cathepsin G, BPI negative

The histopathological features corresponded with anti-VCA (viral capsid antigen) and anti-EBNA (Epstein-Barr virus nuclear antigen) IgG antibodies and EBV-DNA expression in peripheral blood as shown in Table 4. Considering the expanding peripheral blood EBV-DNA expression, the therapy with anti-CD20 was instituted, resulting in complete waning of EBV-DNA copies in peripheral blood. The patient is being regularly monitored by specialists in pediatric immunology and hematology/oncology considering the potential administration of an mTOR (mammalian target of rapamycin) inhibitor, rapamycin. The results of DN T cells and EBV-DNA expression monitoring over time are displayed in Table 5.

**Table 5:** DN T-cells and EBV-DNA monitoring at 2-month intervals.

**No**	**CD3+CD4-CD8- DN in T cells**		**EBV-DNA**
	**%**	**Cells/mcL**	**Copies/mL**
1	6.7	100	86
2	10.2	195	554
3	0	0	20
4	15.2	306	1526 → anti-CD20
5	17.0	413	0
6	13.8	372	0

## Discussion

Chronic lymphadenopathy and splenomegaly reflecting lymphoproliferation along with cytopenia in children pose a clinical and diagnostic challenge and exemplifies the need for a thorough differential diagnosis, including ALPS and clinically overlapping immunodeficiencies (16–20), malignancies (20, 21), infections (15, 20), autoimmune (22), and metabolic disorders (23, 24). Furthermore, the recommended diagnostic algorithm of ALPS developed by NIH experts (6) introduces required and accessory clinical and laboratory immunological as well as genetic criteria, engaging primarily the relative count of TCR alpha/beta CD3+CD4-CD8- T cells, levels of IL-10, IL-18, vitamin B12 as biomarkers, and examination of FAS-mediated apoptosis and soluble FASL level. The ESID diagnostic criteria for ALPS are guided by the discriminatory laboratory markers but their construction does not include genetic testing and leaves more space for the individual disease phenotype (9).

However, the analyses of the predictive value of biomarkers and double-negative T cells in patients with lymphoproliferation and autoimmune cytopenia proved their limited efficiency as correlates with germline *FAS* mutations and the role of lymphocyte apoptosis assays as a key diagnostic marker for ALPS (8) and sFASL assessment as the initial diagnostic step were underscored (25). A combination of autoimmune cytopenias and hypergammaglobulinemia was also shown to have a predictive value of ALPS in patients with lymphoproliferation and elevated double-negative T cells (26). In this setting, *in vitro* examination of Fas-mediated lymphocyte apoptosis requires a multistep, labor-intensive assay with mitogen activation, IL-2 stimulated culture, and anti-Fas IgM monoclonal antibody exposure, which is a fine, but expensive test with limited availability and unsuitability for routine clinical use (26, 27). Instead, despite the highly variable expressivity of ALPS mutations, the increasing number of achievements in immunogenetics allow for an accurate molecular genetic diagnosis in more than 70% of affected patients and thereby enable establishing a definitive clinical diagnosis of ALPS (26). Given these challenges to be able to accurately diagnose ALPS, we sought to determine, which key predictive markers are found in our patient studied. Chronic non-malignant lymphoproliferation, cytopenia, polyclonal hypergammaglobulinemia, elevated TCR alpha/beta CD3+CD4-CD8- T cells, biomarker Il-10 and cobalamin levels, though their variable sensitivity and specificity as sole or combined indicators (8, 25, 27, 28), were observable thereby fulfilling ESID diagnostic criteria (9). Confirming germline *FAS* pathogenic variant in our patient, the revised NIH criteria for the definitive diagnosis were also accomplished (6).

According to the NIH criteria, the differential diagnosis in pediatric patients with lymphoproliferative disorders presenting clinical phenotypes overlapping with ALPS also requires tissue biopsy and distinctive histopathology to exclude several distinguishable autoimmune, malignant, and infectious disorders. This spectrum encompasses Castleman disease which shares a common mTOR activation pathway with ALPS (29–31), Kikuchi-Fujimoto disease (32), Rosai-Dorfman disease (33), RALD (7), X-linked lymphoproliferative disease (XLP) (34), or caspase eight deficiency syndrome (CEDS) (35). In both ALPS and CAEBV, the histological features of the biopsied lymph node may be similar. The most prominent finding in an EBV infection is the marked paracortical expansion of small lymphocytes, immunoblasts, and plasma cells. Many of the paracortical lymphocytes express markers of associated with cytotoxicity, such as perforin, TIA1 (cytotoxic granule-associated RNA binding protein, associated with apoptosis), CD57, and demonstrate a high proliferative index. In an EBV infection, the presence of an EBER-positive lymphocyte subset and reactive T cells expressing the gamma/delta TCR are common histological features. In our patient, the histopathology of the biopsied lymph node was not confirmatory for ALPS, but instead, it showed features of CAEBV with EBER-positive cells. This indicates potential diagnostic pitfalls associated with an EBV infection in face of ALPS favoring hematology, flow cytometric, biomarkers, and genetic approach.

As an impaired antiviral signaling and a defective controlling of an EBV infection may play an adjunctive role in the immunopathogenesis of these syndromes, yet the link between the EBV infection and the development of lymphoproliferative disease due to malfunctioning apoptosis pathway in ALPS requires further studies (15).

CAEBV is a life-threatening EBV-associated T or NK cell lymphoproliferative disease, characterized by an increased EBV viral load in peripheral blood and expansion of EBV-infected T and/or NK cells (36–39). The clinical course of CAEBV is heterogeneous, ranging from an inactive course and stable patient condition and occasionally a self-limiting disease to an aggressive course and with the fatal outcome due to hemophagocytic lymphohistiocytosis, multiple organ failure, or progression to leukemia or lymphoma (39–41). The immunopathogenesis of CAEBV is complex showing features of both malignancy and immunodeficiency (36). The systemic form of CAEBV is clinically characterized by lymphadenopathy, splenomegaly, hepatomegaly, and fevers. Additional CAEBV complications may include pulmonary, neurological, digestive, cardiovascular, dermal, and ocular disorders (39–41), which may also constitute overlapping symptomatology of ALPS (26) making the clinical presentation of lymphoproliferation misleading. Finally, cytopenia, hypergammaglobulinemia, autoimmunity, an elevated IL-10 production as a result of Th2-skewed response essential for ALPS pathogenesis, with an increased risk of malignant lymphoma are further common features attributable to both ALPS and CAEBV. In children, T-cell CAEBV has been more often reported than in adults, with a less aggressive clinical course. It requires a differential diagnosis with either NK-cell or B-cell CAEBV as well as other EBV-related lymphoproliferative diseases to establish an appropriate therapeutic approach. Careful monitoring and observation are required for early recognition of HLH or lymphoma in affected patients (40–45).

Several inborn errors of immunity are particularly burdened with a high risk of EBV-induced immune dysregulation and malignant lymphoproliferation. Mutations in genes encoding costimulatory molecules, involved in T-cell mediated immunity, such as members of the tumor necrosis factor (TNF) receptor superfamily (TNFRSF), CD137, CD27, and its ligand CD70, as well as coronin 1A (CORO1A), cytidine triphosphate synthase 1 (CTPS1), IL2-inducible T-cell kinase (ITK), magnesium transporter 1 (MAGT1), Ras guanyl releasing protein 1 (RASGRP1), serine/threonine kinase 4 (STK4), capping protein Arp 2/3 and myosin-I linker 2 (CARMIL2), SH2 domain-containing protein 1A (SH2D1A), X-linked inhibitor of apoptosis (XIAP) are associated with extreme susceptibility to the EBV-induced tumorigenesis (38).

In conclusion, symptomatology of ALPS may parallel CAEBV thereby posing the risk of misdiagnosis and incorrect therapy. In light of this diagnostic challenge to differentiate between these mutually mimicking disorders, the role of the precise analysis of NIH and ESID criteria, including biomarker approach and genetic molecular investigations as the basis of the definitive diagnosis, needs to be highlighted. Further studies are required to address the issue of the assumptive role of an EBV infection as a triggering immunopathogenetic factor for ALPS.

## Data Availability Statement

The original contributions presented in the study are included in the article/Supplementary Material, further inquiries can be directed to the corresponding author.

## Ethics Statement

Ethical review and approval was not required for the study on human participants in accordance with the local legislation and institutional requirements. Written informed consent to participate in this study was provided by the participants' legal guardian/next of kin. Written informed consent was obtained from the minor(s)' legal guardian/next of kin for the publication of any potentially identifiable images or data included in this article.

## Author Contributions

AS-P was responsible for the conception and design of the study, acquisition, interpretation of data, and drafted the manuscript. EG contributed to the design of the study, acquisition, and interpretation of data. ES helped in the acquisition and analysis of data and drafting the manuscript. JM and AM-K contributed to the diagnosing the patient and interpretation of data. All authors contributed to the article and approved the submitted version.

## Conflict of Interest

The authors declare that the research was conducted in the absence of any commercial or financial relationships that could be construed as a potential conflict of interest.

## Publisher's Note

All claims expressed in this article are solely those of the authors and do not necessarily represent those of their affiliated organizations, or those of the publisher, the editors and the reviewers. Any product that may be evaluated in this article, or claim that may be made by its manufacturer, is not guaranteed or endorsed by the publisher.

## Supplementary Material

Supplementary Figure 1 Timeline showing the clinical course of the disease and results of immunodiagnostic investigations.
