# Supplementary figures and images for: Case Report: Autoimmune Lymphoproliferative Syndrome vs. Chronic Active Epstein-Barr Virus Infection in Children: A Diagnostic Challenge
Source: Front Pediatr. 2021 Dec 30;9:798959. doi: 10.3389/fped.2021.798959 (PMC8757380; doi:10.3389/fped.2021.798959)

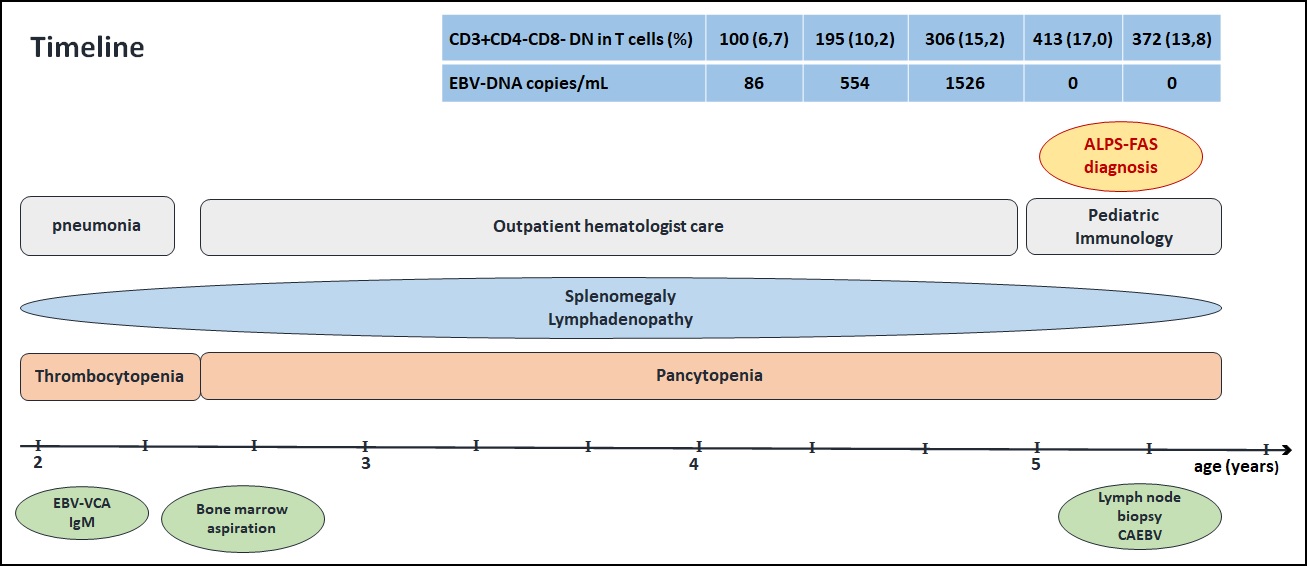

Supplement: Supplementary Figure 1 — Timeline showing the clinical course of the disease and results of immunodiagnostic investigations. [file Image_1.JPEG]
